# Selection and validation of experimental condition-specific reference genes for qRT-PCR in *Metopolophium dirhodum* (Walker) (Hemiptera: Aphididae)

**DOI:** 10.1038/s41598-020-78974-z

**Published:** 2020-12-15

**Authors:** Xinan Li, Peipan Gong, Bingting Wang, Chao Wang, Mengyi Li, Yunhui Zhang, Xiangrui Li, Haifeng Gao, Jiansong Ju, Xun Zhu

**Affiliations:** 1 State Key Laboratory for Biology of Plant Diseases and Insect Pests, Institute of Plant Protection, Chinese Academy of Agricultural Sciences, Yuanmingyuan West Road No.2, Haidian District, Beijing, 100193 China; 2 School of Resource and Environmental Sciences, Henan Institute of Science and Technology, Eastern HuaLan Avenue, Xinxiang, 453003 Henan China; 3 College of Life Science, Hebei Normal University, Road Nan er huan dong No.20, Shijiazhuang, 050024 Hebei China; 4 Institute of Plant Protection, Xinjiang Academy of Agricultural Sciences/Key Laboratory of Integrated Pest Management on Crop in Northwestern Oasis, Ministry of Agriculture and Rural Affairs, Ürümqi, 830091 China

**Keywords:** Reverse transcription polymerase chain reaction, Entomology

## Abstract

*Metopolophium dirhodum* (Walker) (Hemiptera: Aphididae) is one of the most common aphid pests of winter cereals. To facilitate accurate gene expression analyses with qRT-PCR assays, the expression stability of candidate reference genes under specific experimental conditions must be verified before they can be used to normalize target gene expression levels. In this study, 10 candidate reference genes in *M*. *dirhodum* were analyzed by qRT-PCR under various experimental conditions. Their expression stability was evaluated with delta Ct, BestKeeper, geNorm, and NormFinder methods, and the final stability ranking was determined with RefFinder. The results indicate that the most appropriate sets of internal controls were *SDHB* and *RPL8* across geographic population; *RPL8*, *Actin*, and *GAPDH* across developmental stage; *SDHB* and *NADH* across body part; *RPL8* and *Actin* across wing dimorphism and temperature; *RPL4* and *EF1A* across starvation stress; *AK* and *RPL4* across insecticide treatments; *RPL8* and *NADH* across antibiotic treatments; *RPL8*, *RPL4*, *Actin*, and *NADH* across all samples. The results of this study provide useful insights for establishing a standardized qRT-PCR procedure for *M. dirhodum* and may be relevant for identifying appropriate reference genes for molecular analyses of related insects.

## Introduction

The quantitative analysis of target gene expression is an essential part of most molecular studies. Quantitative real-time PCR (qRT-PCR) is a powerful tool for quantifying gene expression, combining improvements in both sensitivity and specificity with efficient techniques for signal detection. It is useful for the quantitative data analysis required for research related to molecular medicine, biotechnology, microbiology, and diagnostics and has become the preferred method for quantifying mRNA^1^. Nevertheless, gene expression analyses are affected by many factors such as the quality of RNA samples, the efficiency of reverse transcription, and PCR efficiency^2,3^. For accurate comparisons of expression levels, the expression data of the genes of interest are normalized against the expression data for a reference gene^4^. Moreover, the reference gene compensates for the above-mentioned limitations^5^. Because housekeeping genes are related to ubiquitous and basic cellular functions, they are considered to be constitutively expressed under diverse conditions^6^. Housekeeping genes, including those encoding *actin*, *glyceraldehyde-3-phosphate dehydrogenase, ribosomal protein*, *18S ribosomal RNA*, *elongation factor 1α* and *heat shock proteins*, have been extensively used as endogenous controls for normalizing real-time PCR data^7–11^. However, several studies have indicated that the expression levels of the reference genes vary under diverse conditions^12–14^. In fact, no single reference gene is appropriate for all experimental conditions. Therefore, evaluating and validating the stability of reference genes under different experimental conditions is critical.

There have recently been several reports regarding reference genes for molecular research on insects, including bumblebee, *Harmonia axyridis*, *Propylea japonica*, *Aphis craccivora* Koch, *Henosepilachna vigintioctomaculata*, *Chilo suppressalis*, *Galeruca daurica*, *Liriomyza trifolii*, *Coccinella septempunctata*, *Phenacoccus solenopsis*, *Lipaphis erysimi*, *Myzus persicae*, *Acyrthosiphon pisum*, and *Megoura viciae*^11,14–27^.

*Metopolophium dirhodum* (Walker) (Hemiptera: Aphididae) is one of the most major aphid pests affecting winter wheat and other cereals worldwide^28–31^. Additionally, *M. dirhodum*, which was first detected in the 1970s, originated in the Holarctic and was subsequently introduced to South America and other regions^32,33^. The *M. dirhodum* nymphs and adults damage cereals by directly feeding on plants, which may result in grain yield losses of 27–30%^34^. Moreover, they damage crops by transmitting several viruses, especially the barley yellow dwarf virus^35^. This aphid has most often been detected in semi-arid regions in South America, South Africa, Australia, and New Zealand, where it damages cereals, including wheat, barley, rye, and oat. A previous study revealed that *M. dirhodum* is the most abundant aphid species on cereals in the continental climate of central Europe^33^. With the technical advances occurring in the post-genomic era, researchers may soon have additional options for studying *M. dirhodum* at the molecular level, which may contribute to the development of improved control measures. Thus, identifying suitable reference genes is important for analyzing the expression of functional genes and for evaluating the efficiency of target gene silencing via RNA interference.

The objective of this study was to identify and evaluate a suite of experimental condition-specific reference genes to normalize target gene expression in *M. dirhodum*. Specifically, we analyzed the following 10 candidate genes: *Actin*, *glyceraldehyde-3-phosphate dehydrogenase* (*GAPDH*), *NADH dehydrogenase* (*NADH*), *arginine kinase* (*AK*), *succinate dehydrogenase B* (*SDHB*), *ribosomal protein L8* (*RPL8*), *18S ribosomal RNA* (*18S*), *elongation factor 1α* (*EF1A*), *ribosomal protein L4* (*RPL4*), and *heat shock protein 68* (*HSP68*). The effects of the following factors on reference gene expression were evaluated: geographic population, developmental stage, body part, wing dimorphism, temperature, starvation stress, and exposure to an insecticide or antibiotic. The results indicate that the best reference genes for analyzing *M. dirhodum* gene expression vary among conditions.

## Results

### Expression levels of candidate reference genes

**Figure 1:**
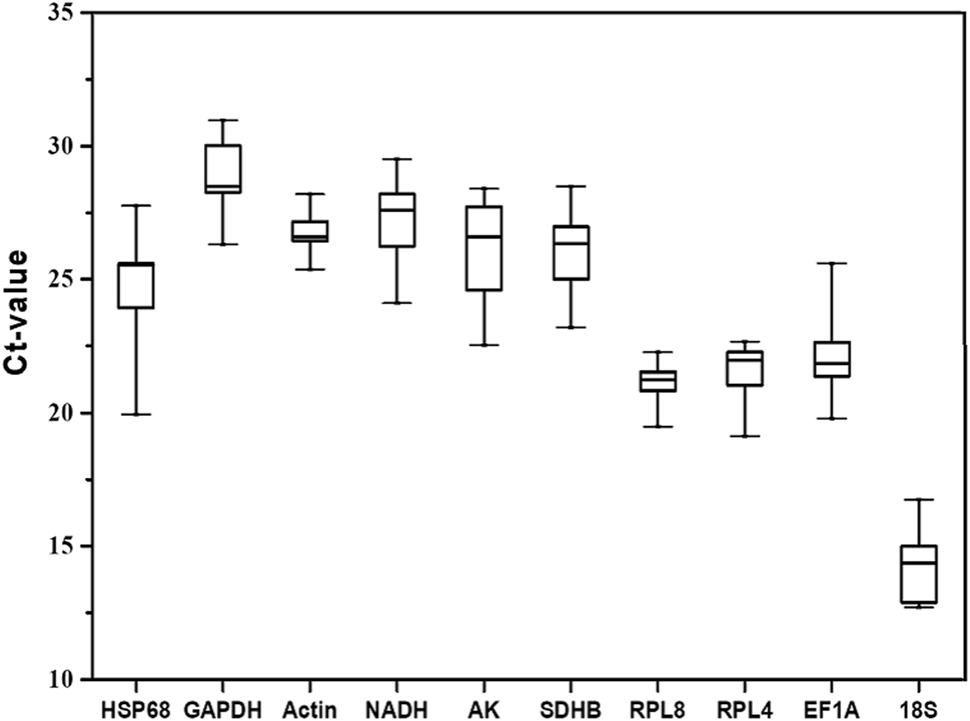
Candidate reference gene expression levels. Candidate reference gene expression levels in the whole *M. dirhodum* sample set are expressed in terms of the threshold cycle number (Ct value). Data are presented as whisker box plots. The box represents the 25th–75th percentiles, the median is indicated by a bar across the box, and the whiskers on each box represent the minimum and maximum values.

To evaluate the expression profiles of the selected candidate genes in all *M. dirhodum* sample sets, mRNA levels were measured for all genes. The gene expression levels varied considerably between Ct values of 12.70 (*18S*) and 30.88 (*GAPDH*) (Fig. 1). Of the 10 analyzed genes, the highest and lowest expression levels were detected for *18S* (mean Ct value of 14.27) and *GAPDH* (mean Ct value of 28.90), respectively. The least variable expression among all samples was observed for *Actin* (mean Ct value ± SD of 26.79 ± 0.42) and *RPL8* (21.10 ± 0.35). In contrast, *HSP68* (24.82 ± 1.86) exhibited the most variable expression in all the tested samples.

### Analysis of gene expression stability

#### Geographic populations

**Figure 2:**
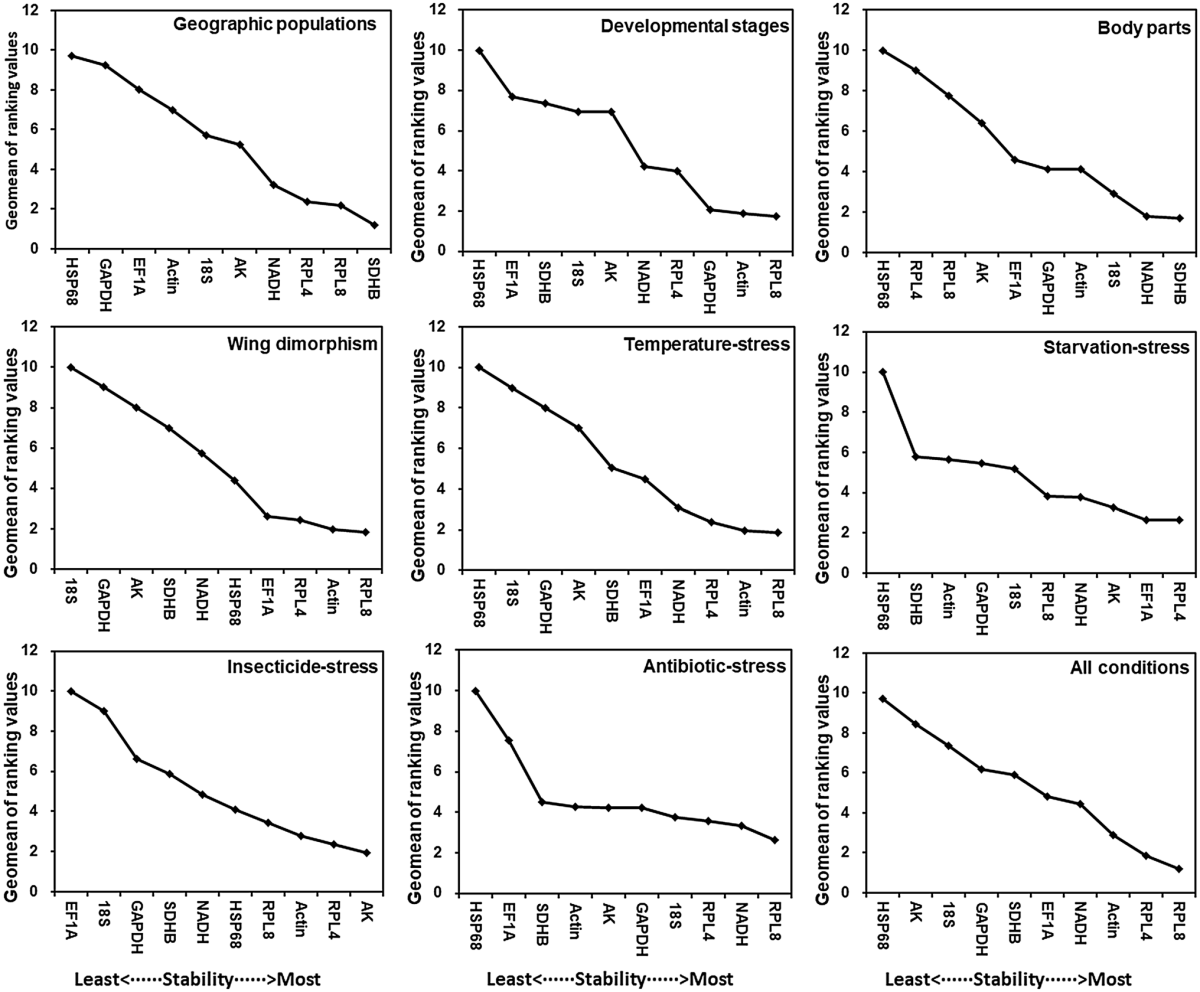
Stability of candidate reference gene expression levels in response to various treatments and conditions. In a RefFinder analysis, decreasing Geomean values correspond to increasing gene expression stability. The Geomean values for the following *M. dirhodum* samples are presented: adult samples from different geographic populations (Geographic population), samples for all developmental stages (Developmental stages), samples for different body parts of wingless adults (Body part), samples for winged and wingless adults (Wing dimorphism), adult samples exposed to different temperatures (Temperature-stress), fed and unfed adult samples (Starvation-stress), adult samples treated with different insecticides (Insecticide-stress), adult samples treated with antibiotic (Antibiotic-stress), and all samples for all treatments (All conditions). The candidate reference genes are as follows: *Actin*, *Actin*; *GAPDH*, *glyceraldehyde-3-phosphate dehydrogenase*; *NADH*, *NADH dehydrogenase*; *AK*, *arginine kinase*; *SDHB*, *succinate dehydrogenase B*; *RPL8*, *ribosomal protein L18*; *RPL4*, *ribosomal protein L4*; *HSP68*, *heat shock protein 68*; *18S*, *18S ribosomal RNA*; and *EF1A*, *elongation factor 1α*.

**Figure 3:**
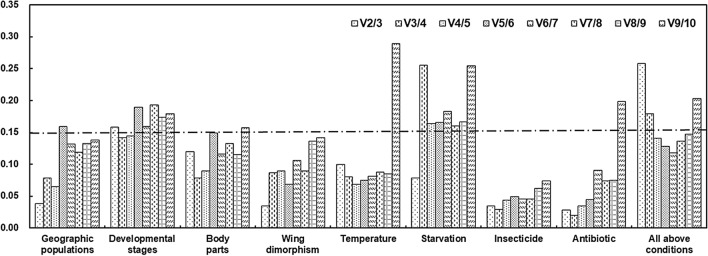
Determination of the optimal number of reference genes for accurate normalization calculated by geNorm. The V_n/n+1_ value indicates the pairwise variation (Y axis) between two sequential normalization factors and determines the optimal number of reference genes required for an accurate data normalization. A value below 0.15 indicates that an additional reference gene will not significantly improve the normalization.

The delta Ct method and the BestKeeper, NormFinder, and geNorm algorithms were used to assess the stability of the candidate reference gene expression levels. The rank order (most to least stable expression) was highly consistent among the four methods. Specifically, *SDHB*, *RPL4*, and *RPL8* were identified as the most stable genes, whereas *HSP68* and *GAPDH* were the least stable genes (Table 1). The RefFinder results for the geographic populations revealed a rank order (most to least stable expression) of *SDHB*, *RPL8*, *RPL4*, *NADH*, *AK*, *18S*, *Actin*, *EF1A*, *GAPDH*, and *HSP68* (Fig. 2). On the basis of the GeNorm analysis, all pairwise variation values were below the 0.15 cut-off value, except for V5/6 (Fig. 3). Moreover, the RefFinder analysis indicated *SDHB* and *RPL8* are required for the normalization of target gene expression levels in different geographic populations.

**Table 1:** Rank order of the *M. dirhodum* candidate reference genes under various experimental conditions.

Experimental conditions	Rank	Delta CT		BestKeeper		NormFinder		GeNorm	
		Gene name	Standard deviation	Gene name	Standard deviation	Gene name	Stability value	Gene name	Stability value
Geographic populations	1	SDHB	0.73	SDHB	0.04	RPL4	0.292	SDHB/RPL8	0.123
	2	RPL4	0.74	RPL8	0.12	SDHB	0.311		
	3	NADH	0.78	NADH	0.13	RPL8	0.439	NADH	0.129
	4	RPL8	0.78	RPL4	0.20	NADH	0.474	RPL4	0.225
	5	AK	0.85	AK	0.29	18S	0.537	AK	0.274
	6	18S	0.90	18S	0.62	AK	0.593	18S	0.507
	7	Actin	0.96	Actin	0.68	Actin	0.707	Actin	0.640
	8	EF1A	1.06	EF1A	0.75	EF1A	0.862	EF1A	0.727
	9	GAPDH	1.38	HSP68	0.84	GAPDH	1.310	GAPDH	0.843
	10	HSP68	1.44	GAPDH	1.08	HSP68	1.366	HSP68	0.962
Development-al stages	1	GAPDH	1.03	RPL8	0.61	GAPDH	0.149	Actin/RPL8	0.461
	2	Actin	1.04	RPL4	0.64	Actin	0.231		
	3	RPL8	1.08	Actin	0.85	RPL8	0.410	GAPDH	0.504
	4	NADH	1.10	SDHB	0.89	NADH	0.458	NADH	0.564
	5	RPL4	1.31	NADH	0.94	RPL4	0.934	RPL4	0.653
	6	18S	1.55	GAPDH	0.96	AK	1.199	EF1A	0.840
	7	AK	1.55	AK	1.40	18S	1.266	18S	0.949
	8	EF1A	1.56	18S	1.47	EF1A	1.301	AK	1.123
	9	SDHB	1.75	EF1A	1.50	SDHB	1.503	SDHB	1.251
	10	HSP68	1.97	HSP68	1.57	HSP68	1.763	HSP68	1.395
Body parts	1	SDHB	0.72	GAPDH	0.24	NADH	0.043	NADH/SDHB	0.085
	2	NADH	0.74	18S	0.28	SDHB	0.043		
	3	18S	0.77	EF1A	0.32	18S	0.168	Actin	0.270
	4	Actin	0.81	SDHB	0.36	Actin	0.447	18S	0.314
	5	EF1A	0.88	NADH	0.42	EF1A	0.568	AK	0.377
	6	AK	0.95	Actin	0.50	GAPDH	0.687	EF1A	0.554
	7	GAPDH	0.98	RPL8	0.61	AK	0.711	GAPDH	0.645
	8	RPL8	1.15	AK	0.66	RPL8	1.056	RPL8	0.757
	9	RPL4	1.25	RPL4	0.71	RPL4	1.162	RPL4	0.832
	10	HSP68	1.59	HSP68	1.23	HSP68	1.557	HSP68	0.983
Wing dimorphism	1	Actin	0.60	Actin	0.06	RPL8	0.027	RPL8/EF1A	0.053
	2	RPL4	0.60	RPL4	0.14	EF1A	0.027		
	3	RPL8	0.62	HSP68	0.19	RPL4	0.039	RPL4	0.087
	4	EF1A	0.64	RPL8	0.19	Actin	0.094	Actin	0.217
	5	HSP68	0.64	NADH	0.23	HSP68	0.332	HSP68	0.309
	6	NADH	0.67	EF1A	0.23	NADH	0.406	NADH	0.344
	7	SDHB	0.94	SDHB	0.51	SDHB	0.889	SDHB	0.458
	8	AK	1.01	AK	0.57	AK	0.982	AK	0.523
	9	GAPDH	1.13	GAPDH	0.74	GAPDH	1.035	GAPDH	0.680
	10	18S	1.41	18S	0.97	18S	1.401	18S	0.827
Temperatures	1	Actin	0.72	RPL8	0.10	RPL4	0.032	Actin/NADH	0.206
	2	RPL8	0.74	RPL4	0.16	RPL8	0.064		
	3	NADH	0.75	SDHB	0.29	Actin	0.141	RPL8	0.280
	4	RPL4	0.78	Actin	0.30	EF1A	0.266	RPL4	0.310
	5	EF1A	0.81	EF1A	0.30	NADH	0.347	EF1A	0.341
	6	SDHB	0.83	NADH	0.32	SDHB	0.397	SDHB	0.386
	7	AK	0.94	AK	0.46	AK	0.502	AK	0.448
	8	GAPDH	1.00	GAPDH	0.60	GAPDH	0.626	GAPDH	0.526
	9	18S	1.11	18S	0.69	18S	0.915	18S	0.595
	10	HSP68	2.92	HSP68	2.18	HSP68	2.885	HSP68	1.059
Starvation-stress	1	RPL4	1.03	18S	0.06	EF1A	0.026	NADH/AK	0.050
	2	EF1A	1.03	Actin	0.33	RPL4	0.026		
	3	RPL8	1.10	GAPDH	0.46	RPL8	0.484	SDHB	0.175
	4	AK	1.23	RPL8	0.78	AK	0.706	RPL4	0.599
	5	NADH	1.26	EF1A	1.02	NADH	0.771	EF1A	0.687
	6	GAPDH	1.31	RPL4	1.05	SDHB	1.034	RPL8	0.790
	7	SDHB	1.40	AK	1.70	GAPDH	1.066	GAPDH	0.930
	8	Actin	1.43	NADH	1.74	Actin	1.280	Actin	1.017
	9	18S	1.77	SDHB	1.89	18S	1.727	18S	1.125
	10	HSP68	2.55	HSP68	2.80	HSP68	2.527	HSP68	1.409
Insecticide-stress	1	RPL4	0.32	HSP68	0.12	AK	0.129	Actin/AK	0.028
	2	AK	0.32	SDHB	0.22	RPL4	0.135		
	3	Actin	0.33	RPL8	0.22	NADH	0.154	RPL8	0.080
	4	RPL8	0.33	RPL4	0.23	Actin	0.167	RPL4	0.102
	5	NADH	0.37	Actin	0.29	GAPDH	0.192	HSP68	0.151
	6	GAPDH	0.39	NADH	0.29	RPL8	0.208	NADH	0.205
	7	HSP68	0.44	AK	0.31	SDHB	0.384	SDHB	0.245
	8	SDHB	0.47	GAPDH	0.50	HSP68	0.388	GAPDH	0.281
	9	18S	0.56	18S	0.69	18S	0.478	18S	0.347
	10	EF1A	0.76	EF1A	0.77	EF1A	0.731	EF1A	0.431
Antibiotic-stress	1	RPL8	0.54	SDHB	0.03	NADH	0.024	GAPDH/18S	0.013
	2	RPL4	0.54	Actin	0.15	RPL8	0.086		
	3	AK	0.54	NADH	0.18	Actin	0.087	AK	0.060
	4	18S	0.58	RPL8	0.47	RPL4	0.350	RPL4	0.071
	5	GAPDH	0.59	RPL4	0.59	SDHB	0.371	EF1A	0.112
	6	NADH	0.63	AK	0.61	AK	0.383	RPL8	0.163
	7	Actin	0.65	18S	0.67	18S	0.484	NADH	0.297
	8	EF1A	0.69	GAPDH	0.68	GAPDH	0.501	Actin	0.370
	9	SDHB	0.76	EF1A	0.76	EF1A	0.646	SDHB	0.439
	10	HSP68	1.99	HSP68	0.95	HSP68	1.987	HSP68	0.749
All above conditions	1	RPL8	1.01	Actin	0.54	RPL8	0.401	RPL8/RPL4	0.421
	2	RPL4	1.03	RPL8	0.54	RPL4	0.497		
	3	NADH	1.09	RPL4	0.82	Actin	0.543	EF1A	0.674
	4	Actin	1.10	18S	0.96	NADH	0.624	NADH	0.747
	5	EF1A	1.15	SDHB	1.00	SDHB	0.723	GAPDH	0.786
	6	GAPDH	1.16	EF1A	1.01	EF1A	0.724	Actin	0.827
	7	SDHB	1.17	GAPDH	1.15	GAPDH	0.752	SDHB	0.868
	8	AK	1.44	NADH	1.16	AK	1.159	AK	0.955
	9	18S	1.51	HSP68	1.46	18S	1.230	18S	1.061
	10	HSP68	2.16	AK	1.56	HSP68	2.019	HSP68	1.281

#### Developmental stage

The delta Ct and NormFinder analyses identified *GAPDH* and *Actin* as the most stable genes. In contrast, the most stable genes were *RPL8* and *RPL4* according to BestKeeper and *Actin* and *RPL8* according to GeNorm. Regardless of the method, *HSP68* was identified as the least stable gene (Table 1). According to the RefFinder results, the rank order (most to least stable expression) for the developmental stages was *RPL8*, *Actin*, *GAPDH*, *RPL4*, *NADH*, *18S*, *AK*, *SDHB*, *EF1A*, and *HSP68* (Fig. 2). The GeNorm analysis revealed that the values for V3/4 were less than the proposed 0.15 cut-off (Fig. 3). The RefFinder analysis indicated *RPL8*, *Actin*, and *GAPDH* are required for normalizing target gene expression levels for the different *M. dirhodum* developmental stages.

#### Body part

The gene expression stability rank order determined with BestKeeper differed from that obtained with the other three methods (Table 1). The BestKeeper algorithm identified *GAPDH* and *18S* as the most stable genes. In contrast, the delta Ct method, NormFinder, and GeNorm identified *NADH* and *SDHB* as the most stable genes. All four analyses indicated *RPL4* and *HSP68* were the least stable genes. The RefFinder results for the different body parts revealed a rank order (most to least stable expression) of *SDHB*, *NADH*, *18S*, *Actin*, *GAPDH*, *EF1A*, *AK*, *RPL8*, *RPL4*, and *HSP68* (Fig. 2). On the basis of the GeNorm analysis, all pairwise variation values were below the 0.15 cut-off value, except for V9/10 (Fig. 3). The RefFinder analysis indicated *SDHB* and *NADH* are required for normalizing target gene expression levels in various *M. dirhodum* body parts.

#### Wing dimorphism

The delta Ct and BestKeeper analyses identified *Actin* and *RPL4* as the most stable genes, whereas both NormFinder and GeNorm identified *RPL8* and *EF1A* as the most stable genes. All four analyses indicated that *18S*, *GAPDH*, *AK*, and *SDHB* were the least stable genes (Table 1). The RefFinder data for the wing dimorphism revealed a rank order (most to least stable expression) of *RPL8*, *Actin*, *RPL4*, *EF1A*, *HSP68*, *NADH*, *SDHB*, *AK*, *GAPDH*, and *18S* (Fig. 2). On the basis of the GeNorm analysis, all pairwise variation values were below the 0.15 cut-off value (Fig. 3). According to RefFinder, *RPL8* and *Actin* are required for normalizing target gene expression levels in wing-dimorphic insects.

#### Temperature-induced stress

The delta Ct method identified *Actin* and *RPL8* as the most stable genes. Both BestKeeper and NormFinder identified *RPL8* and *RPL4* as the most stable genes, whereas GeNorm identified *Actin* and *NADH* as the most stable genes. All four analyses indicated *HSP68*, *18S*, *GAPDH*, and *AK* were the least stable genes (Table 1). The RefFinder data for the different temperatures revealed a rank order (most to least stable expression) of *RPL8*, *Actin*, *RPL4*, *NADH*, *EF1A*, *SDHB*, *AK*, *GAPDH*, *18S*, and *HSP68* (Fig. 2). On the basis of the GeNorm analysis, all pairwise variation values were below the 0.15 cut-off value, except for V9/10 (Fig. 3). The RefFinder analysis indicated *RPL8* and *Actin* are required for normalizing target gene expression levels in *M. dirhodum* exposed to different temperatures.

#### Starvation-induced stress

The delta Ct method and the NormFinder algorithm identified *EF1A* and *RPL4* as the most stable genes and *Actin*, *18S*, and *HSP68* as the least stable genes (Table 1). However, BestKeeper identified *18S* and *Actin* as the most stable genes and *SDHB* and *HSP68* as the least stable genes (Table 1). The GeNorm algorithm identified *NADH* and *AK* as the most stable genes and *Actin*, *18S*, and *HSP68* as the least stable genes (Table 1). The RefFinder results for the starvation treatment revealed a rank order (most to least stable expression) of *RPL4*, *EF1A*, *AK*, *NADH*, *RPL8*, *18S*, *GAPDH*, *Actin*, *SDHB*, and *HSP68* (Fig. 2). The GeNorm analysis indicated that the pairwise variation values for V2/3 were less than the proposed 0.15 cut-off (Fig. 3). The RefFinder analysis indicated *RPL4* and *EF1A* are required for normalizing target gene expression levels in starvation-stressed *M. dirhodum*.

#### Insecticide-induced stress

The delta Ct and NormFinder data revealed *AK* and *RPL4* as the most stable genes, whereas the BestKeeper results identified *HSP68* and *SDHB* as the most stable genes. In contrast, *Actin* and *AK* were the most stable genes according to GeNorm. All four analyses identified *18S* and *EF1A* as the least stable genes (Table 1). The RefFinder data for the insecticide treatment revealed a rank order (most to least stable expression) of *AK*, *RPL4*, *Actin*, *RPL8*, *HSP68*, *NADH*, *SDHB*, *GAPDH*, *18S*, and *EF1A* (Fig. 2). Based on the GeNorm analysis, all the pairwise variation values were below 0.15 cut-off value (Fig. 3). Thus, *AK* and *RPL4* are required for normalizing target gene expression levels in insecticide-treated *M. dirhodum*.

#### Antibiotic-induced stress

The delta Ct method identified *RPL8* and *RPL4* as the most stable genes. The BestKeeper algorithm identified *SDHB* and *Actin* as the most stable genes, whereas NormFinder indicated *NADH* and *RPL8* were the most stable genes. The GeNorm algorithm identified *GAPDH* and *18S* as the most stable genes. All four analyses identified *EF1A*, *SDHB*, and *HSP68* as the least stable genes (Table 1). The RefFinder data for the antibiotic treatment revealed a rank order (most to least stable expression) of *RPL8*, *NADH*, *RPL4*, *18S*, *GAPDH*, *AK*, *Actin*, *SDHB*, *EF1A*, and *HSP68* (Fig. 2). According to the GeNorm analysis, all pairwise variation values were less than the proposed 0.15 cut-off, except for V9/10 (Fig. 3). The RefFinder analysis suggested *RPL8* and *NADH* are required for normalizing the target gene expression levels in antibiotic-treated *M. dirhodum*.

### Overall ranking of *M. dirhodum* candidate reference genes

An examination of the candidate reference gene expression stability for all treatments and conditions with the four methods used in this study produced similar rank orders, with *RPL4* and *RPL8* identified as the most stable genes and *AK*, *18S*, and *HSP68* revealed as the least stable genes (Table 1). The RefFinder results for all treatments and conditions revealed a rank order (most to least stable expression) of *RPL8*, *RPL4*, *Actin*, *NADH*, *EF1A*, *SDHB*, *GAPDH*, *18S*, *AK*, and *HSP68* (Fig. 2). The GeNorm analysis indicated that the pairwise variation values for V4/5 were less than the proposed 0.15 cut-off (Fig. 3). Thus, an analysis of all treatments and conditions suggested that *RPL8*, *RPL4*, *Actin*, and *NADH* are suitable internal reference genes for normalizing target gene expression levels in *M. dirhodum*.

## Discussion

There are several reports describing the application of qRT-PCR assays to clarify the gene expression levels associated with diverse biological processes^36–39^. Reference genes used for molecular investigations can influence the accuracy of target gene expression levels^6,40–42^. Therefore, a stable reference gene is an important prerequisite for gene expression investigations. Housekeeping genes, which are constitutively expressed to maintain basic cellular functions, have traditionally been used as internal reference controls^6,10,11^. However, there is no universal reference gene that is stably expressed in all cell and tissue types under different experimental conditions^10,11,43–47^. Therefore, every stable reference gene used to normalize gene expression data should be evaluated under each experimental condition^43,48^.

In this study, qRT-PCR was used to evaluate the expression-level stability of 10 candidate reference genes in *M. dirhodum* across specific conditions. The best reference genes varied among conditions. Specifically, *RPL8* (mean Ct value ± SD, 21.10 ± 0.35) and *Actin* (26.79 ± 0.42) had the least variable expression levels, whereas *HSP68* (24.82 ± 1.86) produced the most variable expression levels among the examined candidate reference genes (Fig. 1). Similarly, *RPL8*, *RPL4*, and *Actin* were the most stable reference genes, whereas *HSP68* and *18S* were the least stable reference genes under most conditions (Fig. 2).

Ribosomal proteins (RPs), which are the principal components of ribosomes, are one of the most highly conserved proteins in all life forms. Earlier research proved that RP-encoding genes are among the most stably expressed reference genes, and have been widely used to normalize gene expression levels in insect molecular investigations during the past 10 years^49^. For example, in *Bradysia odoriphaga*^50^, *RPS15* was the most stably expressed gene in response to various temperature treatments. However, another study indicated that the expression levels of RP-encoding genes may vary under some conditions^49^. Moreover, *RPS20* was detected as the least stably expressed gene for analyzing *Plutella xylostella* geographic populations as well as the effects of the temperature, photoperiod, and insecticides^10^. Consistent with these earlier findings, we identified *RPL8* as the most stable gene in *M. dirhodum* across various conditions (except for analyses of different body parts, starvation stress, and insecticide treatments) (Fig. 2). Additionally, *RPL4* was detected as the most stable gene in response to starvation and insecticide treatments, but was also almost the least stable gene during analyses of various *M. dirhodum* body parts (Fig. 2).

*Actin*, which encodes a major structural protein, is important for cell secretion, motility, cytoplasm flow, and cytoskeleton maintenance. Moreover, *Actin* is expressed at various levels in many cell types, and is considered the ideal reference gene for qRT-PCR, which may explain its frequent use^15,26^. For example, it has been used to study the effects of diet on *B*. *odoriphaga* gene expression^50^ and for investigating *M. persicae* gene expression in different tissues and in response to the temperature, photoperiod, and wing dimorphism^26^. However, in *Helicoverpa armigera*, *Actin* was revealed to be the least stable reference gene following temperature and photoperiod treatments^51^. In our study, *Actin* was identified as one of the most stable reference genes for analyzing developmental stages, temperature effects, and wing dimorphism (Fig. 2).

The *GAPDH* gene has been commonly used as a reference gene in the studies of gene expression^7,52,53^. However, unstable *GAPDH* expression has been detected in *Tetranychus cinnabarinus* developmental stages^54^, in the labial glands and fat bodies of *Bombus terrestris* and *Bombus lucorum*^55^, and in various *Sogatella furcifera* body parts^56^. In the current study, *GAPDH* was revealed as a stably expressed candidate reference gene for analyses of developmental stages (Fig. 2). These results imply that the mechanism underlying the expression stability of endogenous reference genes is complex. Furthermore, the stability of potential reference genes in different biological samples should be tested prior to their use.

The protein encoded by *EF1A* affects translation by catalyzing the GTP-dependent binding of aminoacyl-tRNA to the acceptor site of the ribosome. The *EF1A* gene was recently used as a reference gene in multiple insect gene expression studies^55,57,58^. Our results suggest that *EF1A* is an appropriate reference gene only for analyzing the effects of starvation stress on *M. dirhodum* gene expression (Table 2).

**Table 2:** Recommended reference genes for *M. dirhodum* under various experimental conditions.

Conditions	Reference gene	Conditions	Reference gene
Population	*SDHB*, *RPL8*	Temperature	*RPL8*, *Actin*
Development stage	*RPL8*, *Actin*, *GAPDH*	Starvation	*RPL4*, *EF1A*
Body part	*SDHB*, *NADH*	Insecticide	*AK*, *RPL4*
Wing dimorphism	*RPL8*, *Actin*	Antibiotic	*RPL8*, *NADH*
All conditions	*RPL8*, *RPL4*, *Actin*, *NADH*		

The *AK* gene encodes the phosphagen kinase in invertebrates, and it has rarely been used as a reference gene^59^. An earlier study revealed that *AK* is the most stably expressed gene in the *B. terrestris* labial gland and fat body^60^. In this study, *AK* was identified as the most stable gene following insecticide treatments (Fig. 2). In *A. pisum*, *SDHB* and *NADH* are reportedly the most stable housekeeping genes in developmental stages and in response to various temperatures^11^. However, we determined that *SDHB* and *NADH* are the most stable housekeeping genes only during examinations of different *M. dirhodum* body parts (Fig. 2). These results further suggest that reference gene expression stability is influenced by the experimental conditions.

The *18S* rRNA gene is considered to be an ideal reference control because of its relatively stable expression levels^61^. Accordingly, it has been applied in previous studies involving *Lucilia cuprina*^62^, *Rhodnius prolixus*^63,64^, and *Delphacodes kuscheli*^65^. However, in this study, *18S* was revealed as one of the least stable genes in almost all sample sets, implying it is an inappropriate reference gene for *M. dirhodum* (Fig. 2). This observation is consistent with the results of previous studies that indicated *18S* rRNA is not a stable reference gene in *Bactrocera dorsalis* and *Nilaparvata lugens* under specific experimental conditions^66^. It is transcribed by a separate RNA polymerase, which may explain why rRNA is not a suitable reference control^67^. Moreover, the utility of *18S* for normalizing target gene expression levels in a qRT-PCR assay is limited by the potential imbalance between rRNA and mRNA fractions among samples^61^.

The *HSP68* gene, which belongs to the *HSP70* family, encodes a highly conserved chaperone involved in protein assembly, folding, and transport as well as in antigen processing and presentation. The expression of genes encoding HSPs can be affected by high temperatures or other stresses (e.g., due to chemicals)^68^. In the current study, *HSP68* was the least stable gene for all conditions (Fig. 2). In a previous study on *Coleomegilla maculata*, *HSP70* was identified as the most stably expressed gene for sexes, but was the least stably expressed gene for analyses of different tissues, and dsRNA exposure^44^.

It is becoming common for researchers to use multiple reference genes to normalize target gene expression levels in diverse studies because a single gene is usually insufficient for analyzing gene expression^69^. An earlier investigation indicated that too many or too few reference genes may adversely affect the robustness of data normalizations^70^. However, the simultaneous application of multiple reference genes in a given experiment may decrease the probability of biased normalizations. The optimal number of reference genes under specific experimental conditions can be determined with the geNorm algorithm, which calculates the pairwise variation V_n/n+1_ based on the normalization factors NF_n_ and NF_n+1_, with n ≥ 2. If V_n/n+1_ is below 0.15, n is the optimal number of reference genes. The results of this study indicate that the most appropriate number of reference genes varies under diverse experimental conditions (Fig. 3). This implies that the stability of reference genes must be evaluated before every qRT-PCR experiment.

## Conclusions

To the best of our knowledge, this study is the first to evaluate and validate experimental condition-specific candidate reference genes for *M. dirhodum* gene expression analyses. We identified reference genes applicable for elucidating functional gene expression profiles. In this study, we examined 10 candidate reference genes under diverse conditions. Notably, the stability of candidate gene expression levels in *M. dirhodum* varies depending on the experimental conditions. Moreover, we identified internal reference genes suitable for normalizing and quantifying gene expression in *M. dirhodum* (Table 2). Our findings may be useful for establishing a more accurate and reliable method for normalizing *M. dirhodum* qRT-PCR data. They may also provide the basis for future investigations on RNA interference and gene transcription in *M. dirhodum* and other insect pests.

## Materials and methods

### Insects

Our original *M. dirhodum* colony was collected in Yinchuan (Ningxia), China (38° 48′ 54.78″ N, 106° 30′ 27.93″ E) in 2018. Other colonies were collected in Langfang (Hebei), China (39° 8′ 9.8″ N, 116° 10′ 4.05″ E) and Guiyang (Guizhou), China (26° 0′ 34.08″ N, 106° 35′ 4.35″ E) in 2018. The alive adults were collected in wheat leaves of different plants of these geographic locations and were taken back to the lab to establish population. All the wheat aphid populations were reared on Lunxuan 987 wheat seedlings in a thermostatic chamber maintained at 20 ± 2 °C and 60% relative humidity, with a 16-h light:8-h dark cycle.

### Treatments

#### Geographic population

Insects collected in Yinchuan (Ningxia), Langfang (Hebei), and Guiyang (Guizhou) in 2018 were examined to assess the effects of geography on gene expression. These three locations are separated by more than 1000 km. For each geographic population, three samples of 20 adults were selected, flash frozen in liquid nitrogen, and stored at − 80 °C until total RNA extraction.

#### Developmental stage

Three *M. dirhodum* samples of about 30 first-instar nymphs, 30 second-instar nymphs, 20 third-instar nymphs, 20 fourth-instar nymphs, and 20 adults were collected, flash frozen in liquid nitrogen, and stored at − 80 °C until total RNA extraction.

#### Body part

We used a dissection needle and a tweezer to separate the head, thorax, and abdomen from wingless *M. dirhodum* adults. These body parts as well as whole adult bodies were stored as described earlier.

#### Wing dimorphism

Three samples of 20 winged and wingless *M. dirhodum* adults were collected, flash frozen in liquid nitrogen, and stored at − 80 °C until total RNA extraction.

#### Temperature-induced stress

Potted wheat seedlings infested with *M. dirhodum* were divided into five groups for a 24-h exposure to one of the following five temperatures: 4, 10, 15, 20, and 25 °C. For each temperature, three samples of 20 adults were collected, flash frozen in liquid nitrogen, and stored at − 80 °C until total RNA extraction. None of the temperature treatments were lethal to the aphids.

#### Starvation-induced stress

Adult aphids were placed on moistened filter paper in a Petri dish (9 cm diameter) with no food for a 32-h incubation in a thermostatic chamber (20 ± 2 °C and 60% relative humidity, with a 16-h light:8-h dark cycle). The control (satiated) group comprised aphids able to feed on wheat seedlings in the same conditions. For the control and treatment groups, three samples of 20 adults were collected, flash frozen in liquid nitrogen, and stored at − 80 °C until total RNA extraction. The mortality rate among the starved aphids was approximately 10%.

#### Insecticide-induced stress

The effects of insecticides on the stability of candidate reference genes were assessed in *M. dirhodum* subjected to one of the following three insecticide treatments: imidacloprid (9.87 mg/L), thiamethoxam (122.00 mg/L), and beta-cypermethrin (17.28 mg/L). These concentrations were selected because a bioassay indicated they are 30% to the mortality of the population (LC_30_) (Table S1). Aphids were treated with the insecticides via the leaf dip method^71^. The 1% insecticide stock solutions prepared in acetone were serially diluted with water (containing 0.1% Tween-80) to produce five concentrations. Water (containing 0.1% Tween-80) was used as a control solution. Wheat leaves with *M. dirhodum* were immersed in the prepared solutions for 3–5 s and then placed on moistened filter paper in a Petri dish (9 cm diameter). The samples were incubated for 24 h at 20 ± 2 °C and 60% relative humidity, with a 16-h light:8-h dark cycle. For each concentration, the mortality rate based on three replicates of 30 aphids was calculated. Additionally, for the control and treatment groups, three samples of 20 adults were collected, flash frozen in liquid nitrogen, and stored at − 80 °C until total RNA extraction.

#### Antibiotic-induced stress

The *M. dirhodum* adults were fed a 30% sucrose solution containing 50 µg/mL rifampicin or an antibiotic-free sucrose solution (control) (25 aphids per feeder) for 48 h^72^. For the control and treatment groups, three samples of 20 adults were collected, flash frozen in liquid nitrogen, and stored at − 80 °C until total RNA extraction.

### Total RNA extraction and cDNA synthesis

Total RNA was extracted with Trizol according to the protocol for the TRNzol Universal Reagent (Tiangen, Beijing, China). The ratio of the absorbance at 260 and 280 nm was 1.981–2.121, indicating the extracted RNA was pure. Next, 1 μg RNA was used as the template to synthesize first-strand cDNA with Oligo dT primers using the FastKing gDNA Dispelling RT SuperMix (Tiangen) following the manufacturer-recommended protocol. The synthesized cDNA was stored at − 20 °C.

### Primer design and quantitative real-time PCR

A qRT-PCR assay was completed with the Talent qPCR PreMix (SYBR Green; Tiangen) and the CFX Connect Real-Time system (Bio-Rad, Hercules, CA, USA). Details regarding the primers for *EF1A* and *18S* (Table 3) have been published by NCBI. Primers for the other target genes were designed based on our unpublished RNA sequencing data for *M. dirhodum*. The cDNA of each sample was prepared as a 50 ng/μL working solution. The qRT-PCR was completed in a 25-μL reaction volume comprising 12.5 μL 2 × Talent qPCR PreMix, 1 μL forward primer (100 μM), 1 μL reverse primer (100 μM), 1 μL cDNA working solution, and 9.5 μL RNase-Free ddH_2_0. The PCR program was as follows: 95 °C for 5 min; 40 cycles of 95 °C for 30 s and 60 °C for 30 s. For each treatment, standard curves were produced based on a fivefold dilution series of cDNA as a template according to the linear regression model. The fixed threshold in this study was set to 500 to obtain all the threshold cycle (Ct) values of tested candidate reference genes. The qRT-PCR analyses were completed with three biological replicates and three technical replicates.

**Table 3:** Functions, primer sequences, and amplicon characteristics of the candidate reference genes analyzed in this study.

Gene symbol	Gene name	Gene ID	(Putative) Function	Primer sequences(5′-3′)	^a^L (bp)	^b^E (%)	^c^R^2^
*Actin*	*Actin*	TR9961|c1_g1	Cytoskeletal structural protein	F: CCATGTACCCTGGTATTGC	234	1.106	0.9984
				R: TGTGGGAGGTGATGACTTA			
*GAPDH*	*glyceraldehyde-3-phosphate dehydrogenase*	TR3352|c0_g1	Glycolytic enzyme	F: GGATTACCGACGCTACGC	232	0.977	0.9839
				R: CGCACGCACAAGGATTTA			
*NADH*	*NADH dehydrogenase*	TR12676|c0_g1	Enzyme involved in redox reactions	F: GTCAAACCTGGTGGCTAAA	182	0.941	0.9973
				R: AGTCGTGGCGTCCATACAG			
*AK*	*arginine kinase*	TR3122|c0_g1	Key enzyme for cellular energy metabolism	F: AGTACATAATTTCTACGAGGGT	169	1.014	0.9824
				R: GACATGCCAGTTAAGGGA			
*SDHB*	*succinate dehydrogenase B*	TR11034|c0_g1	protein subunits of succinate dehydrogenase	F: TCACGCCAGATTACCG	221	0.888	0.9998
				R: TAGCTCCATGAACAGAAG			
*RPL8*	*ribosomal protein L18*	TR12462|c0_g1	Structural constituent of ribosome	F: CCACAACCCAGACTCCA	179	0.935	0.9998
				R: TAGGCCAGCAATTACGC			
*RPL4*	*ribosomal protein L4*	TR996|c0_g1	Structural constituent of ribosome	F: AAAGCACCCATCAGACC	155	0.928	0.9961
				R: CGGACACGAGGAATACG			
*HSP68*	*heat shock protein68*	TR7632|c0_g3	Molecular chaperone	F: AAACGGGCTCGGGACA	245	0.955	0.9983
				R: TCGACGGCGGGTGATA			
*18S*	*18S ribosomal RNA*	KT204362.1	Structural constituent of ribosome	F: CGATGATGACGACGTGGTAGT	411	0.904	0.999
				R: ACTACCACGTCGTCATCATCG			
*EF1A*	*elongation factor 1 a*	DQ005156.1	Catalysation of GTP-dependent binding of amynoayl-total RNA to the ribosome	F: GGAACACGCTCTATTGGC	526	0.924	0.9989
				R: CACGACCTACTGGGACTG			

^a^Amplicon length.

^b^qRT-PCR efficiency (based on a standard curve).

^c^Reproducibility of the qRT-PCR.

### Data analysis

The stability of the 10 candidate reference housekeeping genes was evaluated with the geNorm^40^, NormFinder^73^, and BestKeeper^74^ algorithms and the comparative delta Ct method^75^. Finally, we compared and ranked the tested candidate reference genes with the web-based RefFinder analytical tool (https://www.heartcure.com.au/for-researchers).

## Supplementary information

Supplementary Table.
