# Supplementary material for: Selection and validation of experimental condition-specific reference genes for qRT-PCR in Metopolophium dirhodum (Walker) (Hemiptera: Aphididae)
Source: Sci Rep. 2020 Dec 15;10:21951. doi: 10.1038/s41598-020-78974-z (PMC7738536; doi:10.1038/s41598-020-78974-z)
Supplement: Supplementary file 1 — Supplementary Table. [file 41598_2020_78974_MOESM1_ESM.docx]

Selection and validation of experimental condition-specific reference genes for qRT-PCR in *Metopolophium dirhodum* (Walker) (Hemiptera: Aphididae)

Xinan Li^1,2,†^, Peipan Gong^1,†^, Bingting Wang^3^, Chao Wang^1^, Mengyi Li^1^, Yunhui Zhang^1^, Xiangrui Li^1^, Haifeng Gao^4^, Jiansong Ju^3^*, and Xun Zhu^1^*

^1^ Institute of Plant Protection, Chinese Academy of Agricultural Sciences, Beijing, China; State Key Laboratory for Biology of Plant Diseases and Insect Pests, Beijing, China

^2^ College of Plant Protection, Fujian Agriculture and Forestry University, Fuzhou, Fujian, China

^3^ College of Life Science, Hebei Normal University, Shijiazhuang, Hebei, China

^4^ Institute of Plant Protection, Xinjiang Academy of Agricultural Sciences / Key Laboratory of Integrated Pest Management on Crop in Northwestern Oasis, Ministry of Agriculture and Rural Affairs, Urumqi 830091, China

† These authors contribute equally to this work.

* Corresponding Author: Xun Zhu ([zhuxun@caas.cn](mailto:zhuxun@caas.cn)) or Jiansong Ju ([jujiansong@126.com](mailto:jujiansong@126.com))

**Table S1. Insecticide toxicity for** ***M. dirhodum* populations**

| **Insecticides** | **N ^a^** | **Slope ± SE ^b^** | **LC_30_ ^c^** | **r ^d^** |
| --- | --- | --- | --- | --- |
| Imidacloprid | 654 | 0.69±0.23 | 9.87（1.40-69.28） | 0.8634 |
| Thiamethoxam | 550 | 0.78±0.10 | 122.00（72.24-206.04） | 0.9759 |
| Beta cypermethrin | 475 | 0.93±0.09 | 17.28（11.04-27.06） | 0.9856 |

^a^ Number of tested aphids;

^b^ SE = standard error;

^c^ LC_30_ = lethal concentration (30%), which refers to the concentration that will kill 30% of the population in a given period. The LC_30_ is expressed as mg/L and the 95% fiducial limits are indicated in parentheses;

^d^ Linear correlation coefficient of the dose–mortality responses.
